# Changes in renal function after nephroureterectomy for upper urinary tract carcinoma: analysis of a large multicenter cohort (Radical Nephroureterectomy Outcomes (RaNeO) Research Consortium)

**DOI:** 10.1007/s00345-022-04156-3

**Published:** 2022-10-06

**Authors:** Alessandro Tafuri, Michele Marchioni, Clara Cerrato, Andrea Mari, Riccardo Tellini, Katia Odorizzi, Alessandro Veccia, Daniele Amparore, Aliasger Shakir, Umberto Carbonara, Andrea Panunzio, Federica Trovato, Michele Catellani, Letizia M. I. Janello, Lorenzo Bianchi, Giacomo Novara, Fabrizio Dal Moro, Riccardo Schiavina, Elisa De Lorenzis, Paolo Parma, Sebastiano Cimino, Ottavio De Cobelli, Francesco Maiorino, Pierluigi Bove, Fabio Crocerossa, Francesco Cantiello, David D’Andrea, Federica Di Cosmo, Francesco Porpiglia, Pasquale Ditonno, Emanuele Montanari, Francesco Soria, Paolo Gontero, Giovanni Liguori, Carlo Trombetta, Guglielmo Mantica, Marco Borghesi, Carlo Terrone, Francesco Del Giudice, Alessandro Sciarra, Andrea Galosi, Marco Moschini, Shahrokh F. Shariat, Marta Di Nicola, Andrea Minervini, Matteo Ferro, Maria Angela Cerruto, Luigi Schips, Vincenzo Pagliarulo, Alessandro Antonelli

**Affiliations:** 1 Department of Urology, University of Verona, Azienda Ospedaliera Universitaria Integrata, Piazzale Stefani 1, 37126 Verona, Italy; 2 Department of Urology, “Vito Fazzi” Hospital, Lecce Piazza Filippo Muratore, 1, 73100 Lecce, Italy; 3 Department of Neuroscience, Imaging and Clinical Sciences, G. D’Annunzio University, Chieti, Italy; 4 Department of Urology, University of Chieti, Chieti, Italy; 5 Department of Urology, University of Florence, Florence, Italy; 6 Department of Urology, Mantua Hospital, Mantua, Italy; 7 School of Medicine, Division of Urology, Department of Oncology, San Luigi Gonzaga Hospital, University of Turin, Orbassano, Turin, Italy; 8 Keck School of Medicine, Institute of Urology, University of Southern California, Los Angeles, CA USA; 9 Department of Urology, Aldo Moro University, Bari, Italy; 10 Department of Surgery, Urology Section, University of Catania, Catania, Italy; 11 Department of Urology, IEO, European Institute of Oncology IRCCS, Milan, Italy; 12 Division of Urology, IRCCS Azienda Ospedaliero-Universitaria di Bologna, Bologna, Italy; 13 Unit of Urology, Department of Surgery, Oncology and Gastroenterology, University of Padua, Padua, Italy; 14 Department of Urology, Foundation IRCCS Ca’ Granda-Ospedale Maggiore Policlinico, University of Milan, Milan, Italy; 15 Urology Unit, San Carlo di Nancy Hospital - GVM Care and Research, Rome, Italy; 16 Department of Urology, Magna Graecia University of Catanzaro, Catanzaro, Italy; 17 Department of Urology, Comprehensive Cancer Center, Medical University of Vienna, Vienna, Austria; 18 Division of Urology, Department of Surgical Sciences - Urology, Città della Salute e della Scienza di Torino - Molinette Hospital, University of Turin, Turin, Italy; 19 Department of Urology, University of Trieste, Cattinara Hospital - ASUGI, Trieste, Italy; 20 Department of Urology, Policlinico San Martino Hospital, University of Genova, Genoa, Italy; 21 Department of Maternal-Infant and Urological Sciences, Sapienza/Policlinico Umberto I, Rome, Italy; 22 Department of Urology, University of Ancona, Ancona, Italy; 23 Division of Oncology, Unit of Urology, Urological Research Institute, IRCCS Ospedale San Raffaele, Milan, Italy; 24 Department of Medical, Oral and Biotechnological Sciences, G. d’Annunzio University of Chieti, Chieti, Italy

**Keywords:** Upper tract urothelial carcinoma, Radical Nephroureterectomy, Acute Kidney Injury, Chronic Kidney Disease

## Abstract

**Purpose:**

To investigate prevalence and predictors of renal function variation in a multicenter cohort treated with radical nephroureterectomy (RNU) for upper tract urothelial carcinoma (UTUC).

**Methods:**

Patients from 17 tertiary centers were included. Renal function variation was evaluated at postoperative day (POD)—1, 6 and 12 months. Timepoints differences were Δ1 = POD-1 eGFR − baseline eGFR; Δ2 = 6 months eGFR − POD-1 eGFR; Δ3 = 12 months eGFR − 6 months eGFR. We defined POD-1 acute kidney injury (AKI) as an increase in serum creatinine by ≥ 0.3 mg/dl or a 1.5 1.9-fold from baseline. Additionally, a cutoff of 60 ml/min in eGFR was considered to define renal function decline at 6 and 12 months. Logistic regression (LR) and linear mixed (LM) models were used to evaluate the association between clinical factors and eGFR decline and their interaction with follow-up.

**Results:**

A total of 576 were included, of these 409(71.0%) and 403(70.0%) had an eGFR < 60 ml/min at 6 and 12 months, respectively, and 239(41.5%) developed POD-1 AKI. In multivariable LR analysis, age (Odds Ratio, OR 1.05, *p* < 0.001), male gender (OR 0.44, *p* = 0.003), POD-1 AKI (OR 2.88, *p* < 0.001) and preoperative eGFR < 60 ml/min (OR 7.58, *p* < 0.001) were predictors of renal function decline at 6 months. Age (OR 1.06, *p* < 0.001), coronary artery disease (OR 2.68, *p* = 0.007), POD-1 AKI (OR 1.83, *p* = 0.02), and preoperative eGFR < 60 ml/min (OR 7.80, *p* < 0.001) were predictors of renal function decline at 12 months. In LM models, age (*p* = 0.019), hydronephrosis (*p* < 0.001), POD-1 AKI (*p* < 0.001) and pT-stage (*p* = 0.001) influenced renal function variation (*ß* 9.2 ± 0.7, *p* < 0.001) during follow-up.

**Conclusion:**

Age, preoperative eGFR and POD-1 AKI are independent predictors of 6 and 12 months renal function decline after RNU for UTUC.

**Supplementary Information:**

The online version contains supplementary material available at 10.1007/s00345-022-04156-3.

## Introduction

Urothelial carcinoma (UC) is the sixth most common malignancy, and upper urinary tract UC (UTUC) accounts for 5–10% of cases [1]. UTUC has an estimated annual incidence of two cases per 100,000, rising in recent years as a result of enhanced detection and improved bladder cancer survival [2]. UTUC has high-mortality, with more than 150,000 deaths per year [1]. Radical nephroureterectomy (RNU), including removal of kidney, entire ureter, and bladder cuff, is the gold standard for high-risk localized UTUC. Low-risk disease, defined as the presence of unifocal, small (< 2 cm), low-grade and superficial tumor, is suitable for kidney-sparing approaches, which provide equal survival outcomes preserving renal function [3].

UTUC patients have an increased risk of chronic kidney disease(CKD) because of age, comorbidities, smoking exposure, and potential impairment of contralateral kidney due to diagnostic procedures or contralateral UTUC [4]. Previous studies showed that CKD may lead to worse overall and cancer-specific survival after treatment for renal cell carcinoma (RCC) as well as UTUC [5, 6]. Despite the benefit of adjuvant chemotherapy in prolonging survival [7] and reducing the risk of disease recurrence in locally advanced UTUC [8], 50% of patients are not eligible for platinum-based protocols due to postoperative renal failure [9, 10]. The identification of patients at risk of significant renal function decline may allow clinicians to better assess kidney-sparing rather than extirpative surgery. Additionally, it may help in developing more appropriate protective strategies such as neoadjuvant treatments or different adjuvant approaches and in a more adequate follow-up schedule for these patients [11, 12]. Clinical factors such as age, cardiovascular disease and low preoperative estimated glomerular filtration rate(eGFR) have been previously associated with renal function decline after RNU [13–15]. However, large-scale and contemporary analyses on predictors associated with renal function impairment are missing. No data have been reported in the recent literature on renal function variation (considering reduction and recovery) after RNU.

The aim of this study is to investigate the prevalence and predictors of renal function variation in a large multicenter cohort of patients who underwent RNU for UTUC.

## Materials and methods

We enrolled 1979 patients from 17 urology tertiary centers affiliated with the Radical Nephroureterectomy Outcomes (RaNeO) Research Consortium, who underwent RNU for UTUC between 1994 and 2020. Only patients who had complete preoperative, postoperative and follow-up renal function data were included. Exclusion criteria consisted of previous or concurrent radical cystectomy, contralateral or metastatic UTUC, and previous renal parenchymal sparing surgery. All patients provided written informed consent for data collection and analysis.

Renal function was evaluated preoperatively, at postoperative day 1(POD-1), at 6 and 12 months. Estimated-GFR, according to Chronic Kidney Disease Epidemiology Collaboration(CKD-EPI) equation [16], was used. A cutoff of 60 ml/min in eGFR was considered to define renal function decline. POD-1 acute kidney injury(AKI) was defined as an increase in serum creatinine by ≥ 0.3 mg/dl or a 1.5–1.9-fold from baseline, according to the Acute Kidney Injury Network(AKIN) classification [17].

The following preoperative data were also retrospectively collected in all centers: gender, age at surgery, *body mass index* (BMI), *American Society of Anesthesiologists*(ASA) classification of physical status, and *Eastern Cooperative Oncology Group*(ECOG) score, comorbidities (presence of *coronary artery disease*[CAD]-, hypertension, hyperlipidemia, diabetes mellitus), smoking exposure, presence of ipsilateral hydronephrosis, preoperative albumin serum level, and preoperative hemoglobin serum level. Perioperative data included operative time, blood loss, intraoperative blood transfusions, POD-1 AKI, POD-1 hemoglobin serum level, postoperative chemotherapy, pathological TNM stage revised according to the AJCC classification system (8th edition) [18], tumor grade, and positive surgical margins(PSMs) rate.

### Statistical analysis

Statistical analysis consisted of three steps. First, descriptive statistics relied on medians and interquartile range (IQR) for continuous variables and on frequencies and percentages (%) for categorical variables. Differences in medians were assessed with the Wilcoxon test, while differences in frequencies were evaluated with the Chi-square test. Post hoc analyses were performed when appropriate and p values were adjusted according to false discovery rate in multiple testing.

Second, we used univariable and multivariable logistic regression models to investigate predictors of eGFR < 60 ml/min at 6 and 12 months and predictors of POD-1 AKI development, according to the data available in the literature [3]. Covariate selection for multivariable logistic regression models was performed with a stepwise selection of covariates in both directions (forward and backward). When appropriate, all the models were forced to include the POD-1 AKI as a covariate. In the final multivariable logistic regression model, only variables that ensure a model performance improvement, as indicated by the Akaike Information Criterion (AIC), were included. Ridgeline plots were used to graphically depict smoothed event of interest probabilities in patients with or without AIC. Third, differences between timepoints were considered as follows: Δ1 = POD-1 eGFR – baseline eGFR; Δ2 = 6 months eGFR – POD-1 eGFR; Δ3 = 12 months eGFR – 6 months eGFR. Linear mixed models, considering the non-independent nature of data and the between and within patients’ variation, were used to test the association between main clinical baseline covariates and renal function variation. The interaction between all covariates and time of observation was tested. A multivariable linear mixed model was built. Covariate selection was performed with a stepwise selection of covariates based on AIC. Covariates with a statistically significant association with eGFR variation or interaction with time of observation were considered for stepwise selection. Multivariable models were built within the overall population and after stratification according to AKI. All statistical analyses were performed using R Statistical Software(version 4.1.0; R Foundation for Statistical Computing, Vienna, Austria). All P values were two-tailed, and a *p* < 0.05 was considered indicative of a statistically significant association.

## Results

After exclusion criteria’ application, 576 patients were included. Among these, 409 (71.0%) were male, 267 (57.5%) had smoking exposure, 224 (46.4%) had ipsilateral hydronephrosis, 269 (50.8%) harbored muscle invasive (MI)-UTUC and 24(4.6%) exhibited PSM. Median preoperative, POD-1, 6 and 12 months eGFR were 62.2 (IQR 48.1–79.9), 48.2 (IQR 37.6–59.0), 49.8 (IQR 37.8–62.2) and 48.8 (IQR 37.8–64.3) ml/min, respectively. At a median follow-up of 27 months (IQR 15–48), median eGFR was 49.5 ml/min (IQR 37.1–65.2). Overall, 239 (41.5%) patients developed POD-1 AKI, 409 (71.0%) had an eGFR < 60 ml/min at 6 months, and 403 (70.0%) had an eGFR < 60 ml/min at 12 months (Table 1).

**Table 1:** Demographics. Continuous covariates are reported as median and interquartile ranges (IQR), categorical covariates are reported as absolute and relative frequencies (%)

Features	Overall (*n* = 576, %)	Features	Overall (*n* = 576)
Age (years)	72.0 (64.0, 79.0)	Estimated blood loss (ml)	170.0 (100.0, 270.0)
Male	409 (71.0%)	Operation time (min)	210.0 (180.0, 255.0)
BMI (kg/m^2^)	26.2 (24.0, 29.0)	Transfusion	51 (8.9%)
BMI category		Intraoperative complications	35 (6.1%)
Normal	163 (35.4%)	Muscle invasive-UTUC	269 (50.8%)
Overweight	88 (19.1%)	pN-stage	
Obese	209 (45.4%)	pN0	171 (29.7%)
Smoking history		pN1	38 (6.6%)
Former	154 (31.2%)	pN2	41 (7.1%)
No	210 (42.5%)	pNx	326 (56.6%)
Yes	130 (26.3%)	Tumor grade	
ASA score > 2	213 (43.8%)	Low grade	436 (75.7%)
ECOG score 0–1	354 (83.1%)	High grade	75 (13.0%)
Hydronephrosis	224 (46.4%)	Unknown	65 (11.3%)
CAD	101 (20.4%)	Positive surgical margin	24 (4.6%)
Hypertension	316 (60.3%)	Main postoperative laboratory	
Hyperlipidemia	199 (38.7%)	I POD HGB (g/dl)	11.2 (10.2, 12.4)
Diabetes	111 (20.4%)	I POD eGFR (ml/min)	48.2 (37.6, 59.0)
Antiplatelet Therapy	121 (24.2%)	eGFR at discharge (ml/min)	50.5 (38.1, 63.0)
Anticoagulant	62 (12.1%)	eGFR at 6 months (ml/min)	49.8 (37.8, 62.2)
Antidiabetic drugs	106 (20.2%)	eGFR at 12 months (ml/min)	48.8 (37.8, 64.3)
Preoperative HGB (g/dL)	13.0 (11.3, 14.2)	I POD AKI	239 (41.5%)
Preoperative Albumin (g/dL)	4.2 (3.8, 33.2)	eGFR < 60 ml/min at 6 months	409 (71.0%)
Preoperative eGFR (ml/min)	62.2 (48.1, 79.9)	eGFR < 60 ml/min at 12 months	403 (70.0%)

*BMI* body mass index, *ASA* American Society of Anesthesiology, *ECOG* Eastern Cooperative Oncology Group, *CAD* Coronary Artery Disease, *HGB* hemoglobin, *eGFR* estimated glomerular filtration rate, *UTUC* upper urinary tract cancer, *POD* postoperative day, *AKI* acute kidney injury

In multivariable logistic regression model, age (Odds Ratio, OR 1.05, 95% CI 1.03–1.08, *p* < 0.001), male gender (OR 0.44, 95% CI 0.25–0.75, *p* = 0.003), POD-1 AKI(OR 2.88, 95% CI 1.78–4.73, *p* < 0.001) and preoperative eGFR < 60 ml/min (OR 7.58, 95% CI 4.46–13.29, *p* < 0.001) were independent predictors of 6 months renal function decline(Table 2). Similarly, preoperative eGFR < 60 ml/min (OR 7.80, 95% CI 4.47–14.12, *p* < 0.001), age (OR 1.06, 95% CI 1.03–1.09, *p* < 0.001), CAD (OR 2.68, 95% CI 1.34–5.61, *p* = 0.007), POD-1 AKI (OR 1.83, 95% CI 1.10–3.08, *p* = 0.02) were independent predictors of 12 months renal function decline (Table 2). Postoperative chemotherapy did not show a statistically significant effect on eGFR < 60 ml/min at 6 and 12 months (OR 0.87 95% CI 0.50–1.57, *p* = 0.636, and OR 0.64 95% CI 0.37–1.13, *p* = 0.116, respectively; data not reported).

**Table 2:** Univariable and multivariable logistic regression models predicting 6 month and 12 months eGFR < 60 ml/min

	6 months 2GFR < 60 ml/min		12 months eGFR z60 ml/min	
	Univariable analysis Odds ratio (95% CI, *p* value)	Multivariable analysis Odds ratio (95% CI, *p* value)	Univariable analysis Odds ratio (95% CI, *p* value)	Multivariable analysis Odds ratio (95% CI, *p* value)
Age	1.08 (1.06–1.10, *p* < 0.001)	1.05 (1.03–1.08, *p* < 0.001)	1.09 (1.06–1.11, *p* < 0.001)	1.06 (1.03–1.09, *p* < 0.001)
Gender (male vs. female)	0.56 (0.36–0.84, *p* = 0.007)	0.44 (0.25–0.75, *p* = 0.003)	0.71 (0.47–1.06, *p* = 0.103)	0.62 (0.35–1.06, *p* = 0.083)
BMI		–		
Overweight vs. normal	1.25 (0.71–2.26, *p* = 0.450)	–	0.95 (0.54–1.67, *p* = 0.852)	–
Obese vs. normal	0.94 (0.60–1.46, *p* = 0.776)	–	0.86 (0.55–1.33, *p* = 0.501)	–
ASA score (> 2 vs. ≤ 2)	2.21 (1.47–3.38, *p* < 0.001)	–	1.73 (1.17–2.59, *p* = 0.007)	–
Hydronephrosis (yes vs. no)	1.22 (0.82–1.82, *p* = 0.322)	–	1.22 (0.83–1.81, *p* = 0.314)	–
CAD	1.70 (1.02–2.94, *p* = 0.047)	–	2.51 (1.46–4.53, *p* = 0.001)	2.68 (1.34–5.61, *p* = 0.007)
Hypertension	1.69 (1.16–2.48, *p* = 0.007)	–	1.18 (0.81–1.72, *p* = 0.384)	–
Hyperlipidemia	0.99 (0.68–1.47, *p* = 0.976)	0.73 (0.45–1.17, *p* = 0.191)	0.86 (0.59–1.27, *p* = 0.453)	0.40 (0.23–0.68, *p* = 0.001)
Diabetes	1.76 (1.08–2.96, *p* = 0.028)	1.49 (0.82–2.77, *p* = 0.200)	1.15 (0.73–1.84, *p* = 0.559)	–
pT-stage (NMI vs. MI)	0.89 (0.61–1.30, *p* = 0.546)	–	1.09 (0.75–1.58, *p* = 0.660)	1.67 (1.02–2.76, *p* = 0.044)
pN-stage				
pN1 vs. pN0	1.41 (0.64–3.34, *p* = 0.411)	2.49 (0.91–7.33, *p* = 0.085)	1.00 (0.48–2.19, *p* = 0.997)	1.53 (0.54–4.43, *p* = 0.424)
pN2 vs. pN0	0.94 (0.46–2.01, *p* = 0.871)	1.02 (0.41–2.65, *p* = 0.972)	0.80 (0.40–1.66, *p* = 0.539)	0.77 (0.31–1.92, *p* = 0.566)
Tumor grade				
Low grade vs. High	0.53 (0.28–0.95, *p* = 0.042)	0.66 (0.29–1.41, *p* = 0.302)	0.40 (0.20–0.74, *p* = 0.005)	0.71 (0.30–1.57, *p* = 0.414)
AKI POD-1	1.55 (1.07–2.26, *p* = 0.022)	2.88 (1.78–4.73, *p* < 0.001)	1.26 (0.88–1.82, *p* = 0.211)	1.83 (1.10–3.08, *p* = 0.020)
eGFR < 60 ml/min preoperatively	6.90 (4.47–10.94, *p* < 0.001)	7.58 (4.46–13.29, *p* < 0.001)	7.09 (4.62–11.19, *p* < 0.001)	7.80 (4.47–14.12, *p* < 0.001)

In multivariable logistic regression model addressing POD-1 AKI, age (OR 1.04, 95% CI 1.02–1.07, *p* < 0.001), (absence of-) preoperative hydronephrosis (OR 0.61, 95% CI 0.39–0.93, *p* = 0.022), preoperative eGFR (OR 1.04, 95% CI 1.03–1.05, *p* < 0.001), CAD (OR 1.89, 95%CI 1.07–3.36, *p* = 0.029), and non-MI-UTUC (OR 1.74, 95% CI 1.14–2.67, *p* = 0.010) were independent predictors of POD-1 AKI development (Supplementary Table 1).

**Fig. 1:**
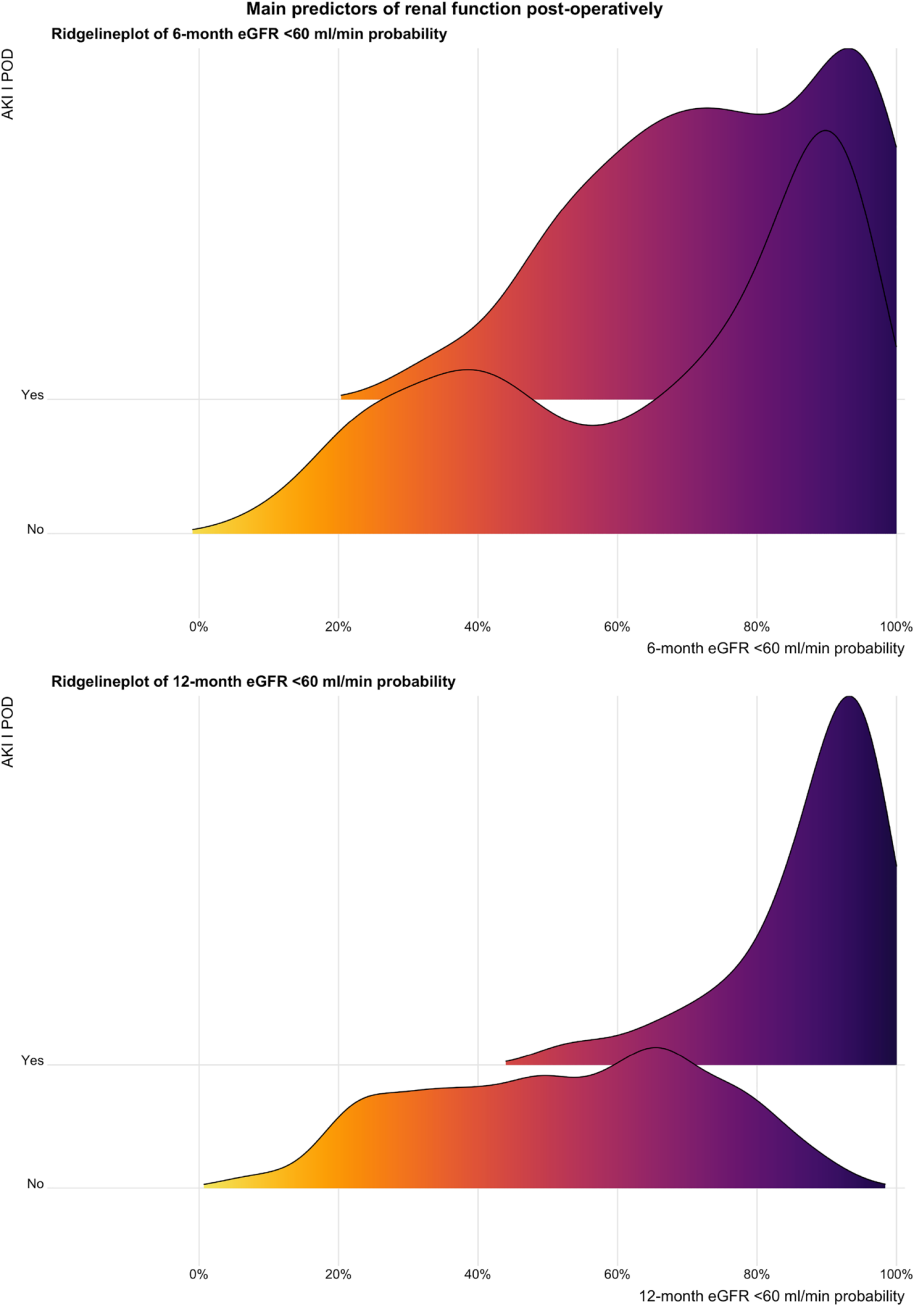
Ridgeline plots show the distribution of 6 and 12 month eGFR < 60 ml/min probability in those with or without AKI. Probabilities were estimated from multivariable logistic regression models. Covariates within the models were selected with stepwise regression according to the best AUC. Figures show that density curves picks are more skewed to the right (higher probabilities) for those who experienced AKI

Ridgeline plots illustrating eGFR < 60 ml/min probabilities distribution showed a detrimental effect of POD-1 AKI on eGFR still at 6 and 12 months. Moreover, ridgeline plots highlighted that the effect of POD-1 AKI on eGFR < 60 ml/min is more pronounced at 12 than 6 months (Fig. 1).

At the previously defined timepoints renal function variation was Δ1 = − 10.2 ml/min (− 25.9; − 2.2), Δ2 = 1.37 ml/min(− 6.2; 9.0), Δ3 = 0.9 ml/min (− 3.6; 5.2 )(Supplementary Fig. 1). Preoperative covariates associated with eGFR variation during follow-up were age (*p* = 0.019) and hydronephrosis (*p* < 0.001). Postoperative covariates associated with eGFR variation during follow-up were POD-1 AKI (*p* < 0.001), and pT-stage (*p* = 0.024) (Table 3, Supplementary Fig. 2). In the entire cohort, ECOG performance status ≤ 1 was associated with a higher renal function reduction during the time-period (*ß* − 2.5 ± 1, *p* < 0.024), and the presence of hydronephrosis was associated with a higher renal function recovery during the time-period (*ß* 9.2 ± 2.2, *p* < 0.001). Among patients with POD-1 AKI, the presence of hydronephrosis (*ß* 2.6 ± 1.3, *p* = 0.045), and ASA score > 2 (*ß* 6.7 ± 3.3, *p* = 0.045) were associated with a lower renal function reduction during the time-period (Supplementary Table 2).

**Table 3:** EGFR variation (ml/min) stratified according to main preoperative covariates

Variables	Median ∆eGFR (IQR)			*p* values		
	Time 1	Time 2	Time 3	Covariates	Time	Interaction
Age				0.009	< 0.001	0.019
< 75 years	– 10.7 (– 28.7, – 2.8)	1.4 (– 6.8, 9.9)	1.0 (– 3.8, 5.5)			
≥ 75 years	– 9.2 (– 23.1, – 1.5)	1.3 (– 5.7, 8.4)	0.6 (– 3.4, 4.4)			
Gender				0.083	< 0.001	0.072
Female	– 9.0 (– 23.6, – 1.0)	– 0.4 (– 7.4, 7.1)	0.8 (– 2.8, 4.7)			
Male	– 10.5 (– 26.8, – 2.8)	2.2 (– 5.9, 10.1)	1.0 (– 3.8, 5.3)			
BMI				0.502	< 0.001	0.455
Normal	– 11.6 (– 26.8, – 2.5)	0.0 (– 7.4, 10.1)	0.0 (– 5.1, 4.4)			
Overweight	– 10.7 (– 27.8, – 2.8)	3.1 (– 6.6, 10.1)	1.6 (– 3.6, 5.4)			
Obese	– 8.7 (– 24.2, – 2.5)	4.1 (– 2.2, 8.4)	1.0 (– 2.7, 5.5)			
ECOG-PS				0.013	< 0.001	0.174
≤ 1	– 10.7 (– 26.4, – 2.8)	1.7 (– 5.4, 9.6)	1.4 (– 3.4, 5.1)			
> 1	– 5.6 (– 18.4, 0.1)	5.1 (– 0.7, 11.7)	1.0 (– 3.2, 5.7)			
ASA score				0.052	< 0.001	0.129
≤ 2	– 10.7 (– 28.3, – 3.1)	3.3 (– 4.9, 11.3)	1.0 (– 3.7, 5.1)			
> 2	– 8.5 (– 23.2, – 0.4)	0.5 (– 7.2, 7.6)	1.0 (– 3.1, 5.3)			
Hydronephrosis				< 0.001	< 0.001	< 0.001
No	– 15.6 (– 29.5, – 4.2)	1.9 (– 6.8, 10.7)	1.7 (– 3.6, 5.2)			
Yes	– 5.5 (– 18.0, – 0.6)	2.1 (– 4.8, 8.2)	0.9 (– 2.7, 4.9)			
CAD				0.713	< 0.001	0.735
No	– 10.0 (– 26.1, – 2.3)	1.3 (– 6.0, 9.6)	0.9 (– 3.2, 5.2)			
Yes	– 10.8 (– 24.1, – 2.8)	2.8 (– 6.6, 7.3)	0.0 (– 4.9, 4.2)			
AKI				< 0.001	< 0.001	< 0.001
No	– 3.2 (– 8.4, 1.8)	– 1.1 (– 9.9, 5.9)	0.5 (– 4.5, 4.6)			
Yes	– 29.3 (– 37.0, – 20.4)	5.0 (– 0.5, 13.2)	1.7 (– 3.0, 5.6)			
pT-stage				0.001	< 0.001	0.024
NMI-UTUC	– 12.8 (– 30.7, – 2.8)	1.3 (– 6.6, 8.4)	0.9 (– 4.0, 5.2)			
MI-UTUC	– 6.9 (– 22.1, – 1.9)	1.9 (– 6.1, 10.6)	1.1 (– 3.2, 4.9)			
pN-stage				0.344	< 0.001	0.427
pN0	– 8.1 (– 23.5, 0.0)	0.8 (– 7.4, 7.4)	1.6 (– 2.7, 4.9)			
pN1	– 4.7 (– 12.2, – 1.9)	3.1 (– 5.0, 7.0)	2.1 (– 1.0, 6.9)			
pN2	– 10.4 (– 31.8, – 4.9)	5.2 (– 2.8, 13.0)	0.5 (– 2.7, 5.1)			
Tumor grade				0.747	< 0.001	0.361
High grade	– 9.6 (– 21.9, – 0.4)	– 2.0 (– 9.6, 5.1)	0.0 (– 5.7, 4.2)			
Low grade	– 10.1 (– 26.1, – 2.6)	3.0 (– 4.9, 10.3)	1.2 (– 3.3, 5.3)			
Margin status				0.020	< 0.001	0.039
Negative	– 10.2 (– 26.2, – 2.6)	1.9 (– 6.1, 9.4)	1.0 (– 3.4, 5.2)			
Positive	– 2.7 (– 8.2, 2.8)	– 2.7 (– 6.8, 5.3)	0.0 (– 3.0, 3.4)			
Complications				0.993	< 0.001	0.958
None	– 10.2 (– 25.7, – 2.3)	1.4 (– 6.1, 9.0)	0.9 (– 3.5, 5.0)			
At least one	– 10.6 (– 27.1, 0.4)	0.0 (– 8.1, 10.1)	0.9 (– 4.1, 5.9)			

The eGFR variation was calculated as the difference between each timepoint and the previous timepoint (i.e., Time 1 = eGFR POD I – eGFR preoperative; Time 2 = eGFR at 6 month – eGFR POD I)

## Discussion

We investigated the predictors of postoperative renal function changes in a large multicenter cohort of patients who underwent RNU for UTUC. We found that age, POD-1 AKI and preoperative eGFR were independent predictors of eGFR < 60 ml/min at 6 months after surgery. Similarly, age, CAD, T-stage, POD-1 AKI, and preoperative eGFR were predictors of an eGFR < 60 ml/min at 12 moths after surgery. Additionally, examining the renal function trend over 12 months, patients with ECOG performance status ≤ 1 had a higher renal function reduction, while the presence of hydronephrosis was associated with lower renal function reduction. Our results are in line with those coming from smaller populations involved in the most recent studies. Faba et al. and Hashimoto et al. in retrospective cohorts of 138 and 110 patients, respectively, found that low preoperative eGFR, age, and the absence of hydronephrosis were predictive factors for impaired postoperative renal function after RNU for UTUC [19, 20]. Song et al. showed that high-BMI, low preoperative eGFR, and low contralateral kidney volume were significantly associated with new-onset CKD in 135 patients who underwent RNU [21]. In 2016, Singla et al. in a cohort of 135RNU patients found that after a median follow-up of 28.6 months, patients without hydronephrosis (53%) experienced a greater decline in eGFR following RNU [22]. These findings suggest that previously established contralateral compensatory kidney hypertrophy due to hydronephrosis of the ipsilateral urinary tract facilitates the compensatory role of the remnant solitary kidney.

Interestingly, in 2018, Lee et al. investigated predictors of renal function recovery in a cohort of 118 RNU patients. Half of those with preoperative eGFR < 60 ml/min achieved eGFR recovery within the first 3 years after RNU, and homolateral hydronephrosis was a significant predictor of renal function recovery [23]. The authors commented that prior to definitive surgical intervention, contralateral kidney compensation has begun, and it has facilitated this renal functional recovery along the time [23].

Considering performance status, it has been already demonstrated that ECOG-PS > 1 is associated with worse oncological outcomes and high-grade complications’ rate after RNU for UTUC [24, 25]. We found ECOG > 1 was associated with lower renal function recovery after surgery. This may reflect a general deficiency status including a lower nephrons’ reserve [26], as confirmed in our population where patients with ECOG-PS > 1 had lower preoperative eGFR compared with ECOG-PS ≤ 1 population [ 50.86 (IQR 39.12–64.50) vs. 63.93 86 (IQR 51.79–80.76) ml/min, (*p* < 0.001); data not shown].

In our study, postoperative chemotherapy did not show a statistically significant effect on eGFR at 6 and 12 months. However, the exact timing of postoperative chemotherapy (immediately after surgery or at recurrence or for disease persistence) was not available for most of the patients. Therefore, we could not include this covariate in multivariable analyses, and its effect on renal function should be elucidated in future studies.

We identified a specific role for POD-1 AKI in renal function decline after RNU. We found that 41.5% of patients experienced AKI on the first POD. In the multivariable model, POD-1 AKI was predicted by absence of hydronephrosis, non-muscle invasive disease and higher preoperative eGFR. In those patients, RNU has a great impact on the residual kidney function, and AKI contributed to a higher probability of developing a 6 and 12 months eGFR reduction. Considering the eGFR trend in AKI population, the presence of hydronephrosis, ASA score > 2 was associated with lower renal function reduction during the time (12 months). Although POD-1 AKI was previously largely investigated after surgery for renal cancer [27, 28], its role was un-investigated after RNU surgery in large-cohort studies. A recent small retrospective single-cohort study investigated how POD-1 AKI influences eGFR reduction, and firstly showed that it was a strong predictor of renal function decline in patients who underwent RNU for UTUC [29]. The authors found that POD-1 AKI affected short- and middle-term renal function impairment, indicating that every effort should be made to prevent POD-1 AKI.

Our findings may have important clinical implications. After RNU for UTUC, patients may require adjuvant chemotherapy for advanced stage or disease progression. Only 50% of patients are still eligible for platinum-based protocols, due postoperative renal function failure [8–10] which effects oncological outcomes as recently demonstrated [5]. Early identification of patients at high risk of eGFR reduction after extirpative surgery, for whom adjuvant therapy is no longer feasible, may be addressed by neoadjuvant regimens, resulting in an increase in survival [11]. Conversely, ineligible patients for neoadjuvant therapy have an increased risk of developing renal function decline after RNU and should be treated with kidney-sparing surgery, reducing the morbidity associated with radical surgery without compromising oncological outcomes [30].

The present investigation is retrospective and suffers of its inherent bias. Specifically, the initial cohort coming from 17 tertiary centers was strongly reduced after inclusion of patients with all available interest data only (from1979 to 576 patients).

Our results are innovative and coming from one of the largest populations investigated. We showed that UTUC patients having a nephron reserve before surgery are at high risk to develop a renal function variation after RNU. We also found that POD-1 AKI is a strong predictor of renal function decline. Therefore, a dedicated perioperative management with avoidance of potentially nephrotoxic agents, close monitoring of serum creatinine and urine output should be adopted. Anesthesiologist may contribute to renal damage prevention by reducing the reduction in renal blood and renal hypoxia and preventing hypotension during surgery [31]. When UTUC patients are counseled before treatment, the risk of renal function decline should be extensively explained.

## Conclusion

Age, preoperative eGFR and POD-1 AKI are independent predictors of renal function decline at 6 and 12 months after RNU for UTUC. Identifying patients at high risk of renal function decline after RNU allows to provide a correct perioperative patients’ management.

## Supplementary Information

Below is the link to the electronic supplementary material.

**Supplementary Fig. 1** eGFR variations at the defined timepoints: baseline, postoperative day 1, 6 months, and 12 months. Differences between each timepoint and the previous one were tested with the Wilcoxon test after adjusting for multiple hypothesis testing according to the false discovery rate method (PDF 217 KB)

**Supplementary Fig. 2** eGFR variation according to clinically meaningful covariates. The eGFR variation was calculated as the difference between each timepoint and the previous timepoint (∆1 = eGFR POD I – eGFR preoperative; ∆2 = eGFR at 6 month – eGFR POD III, ∆3 = eGFR 12 month – eGFR 6 month). Differences between each group were evaluated with the Wilcoxon test after adjusting for multiple hypothesis testing according to the false discovery rate method (PDF 844 KB)

Supplementary file3 (DOCX 21 KB)

Supplementary file4 (DOCX 21 KB)
