# Supplementary figures and images for: Changes in renal function after nephroureterectomy for upper urinary tract carcinoma: analysis of a large multicenter cohort (Radical Nephroureterectomy Outcomes (RaNeO) Research Consortium)
Source: World J Urol. 2022 Oct 6;40(11):2771–9. doi: 10.1007/s00345-022-04156-3 (PMC9617815; doi:10.1007/s00345-022-04156-3)

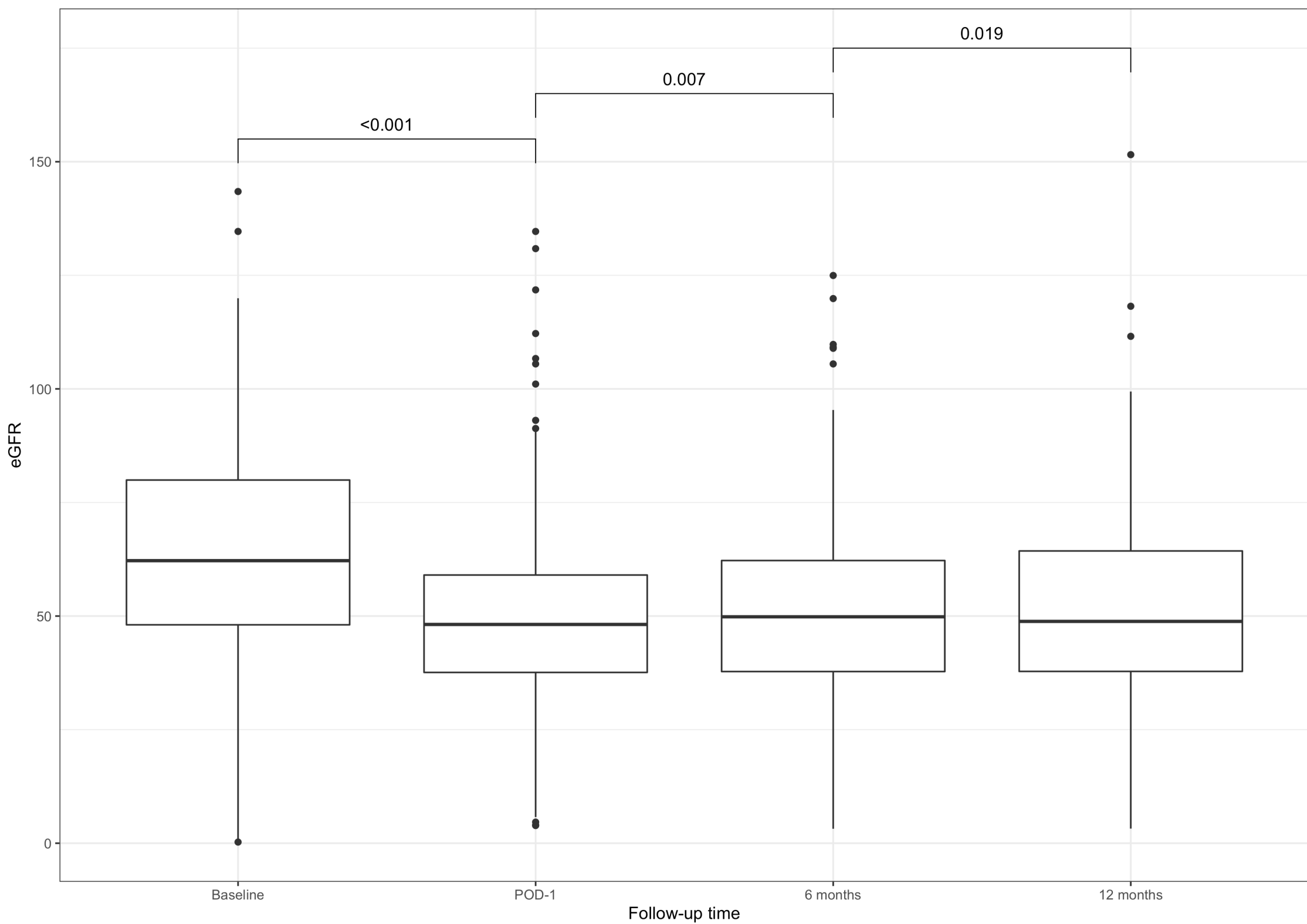

Supplement: Supplementary file 1 — Supplementary Fig. 1 eGFR variations at the defined timepoints: baseline, postoperative day 1, 6 months, and 12 months. Differences between each timepoint and the previous one were tested with the Wilcoxon test after adjusting for multiple hypothesis testing according to the false discovery rate method (PDF 217 KB) [file 345_2022_4156_MOESM1_ESM.pdf]

# eGFR variation

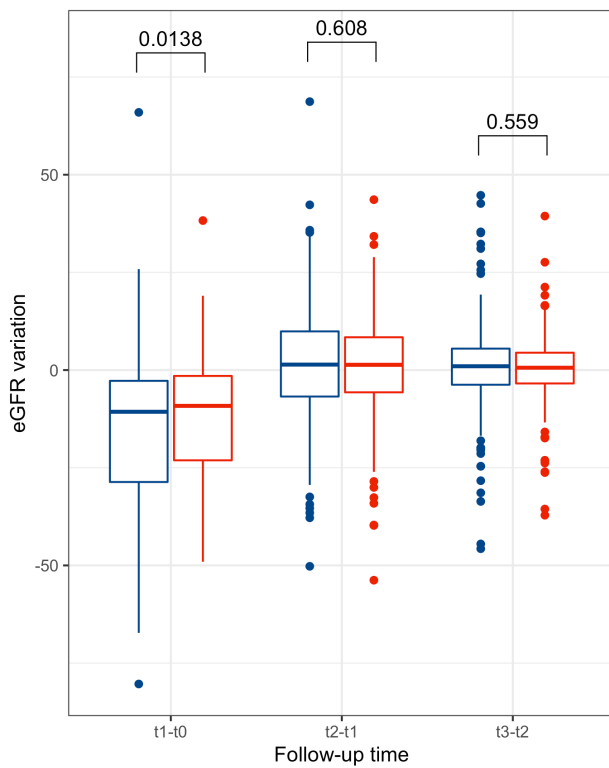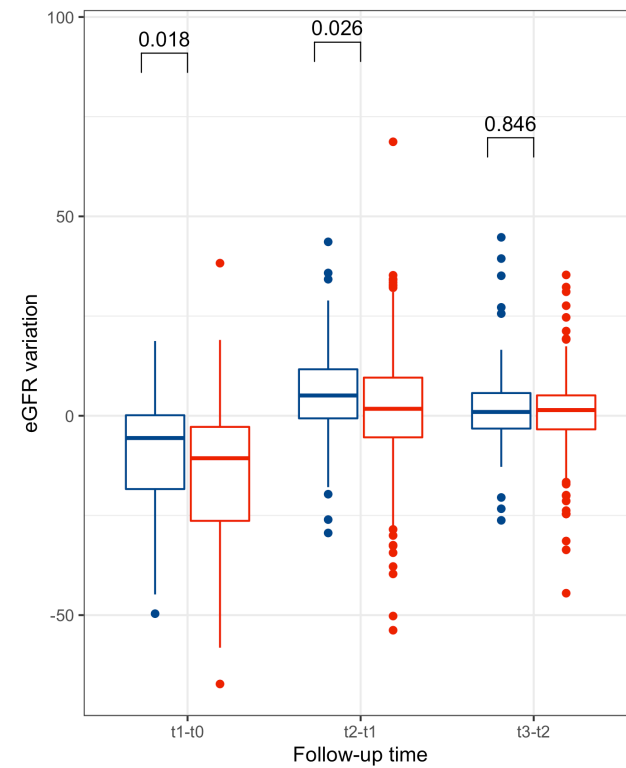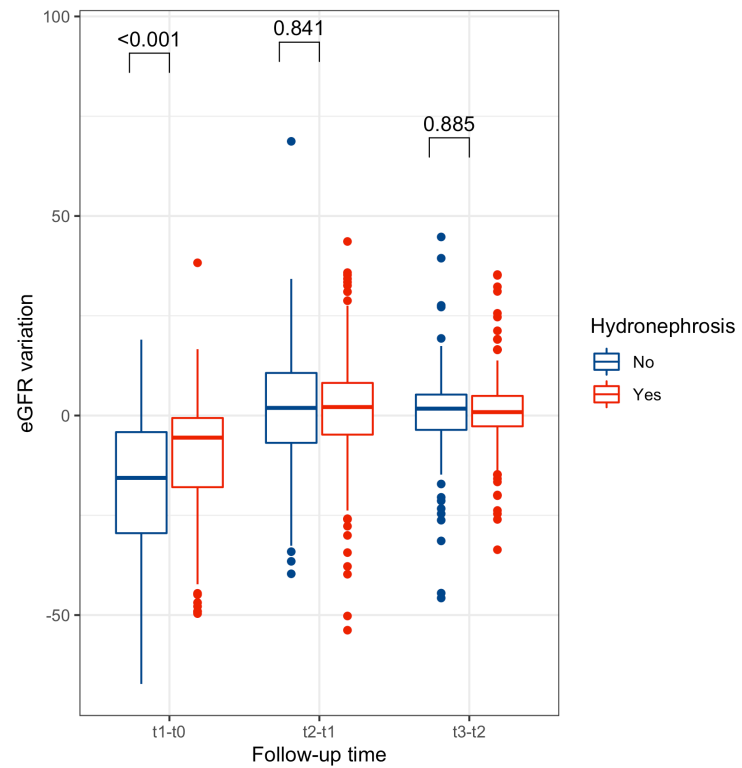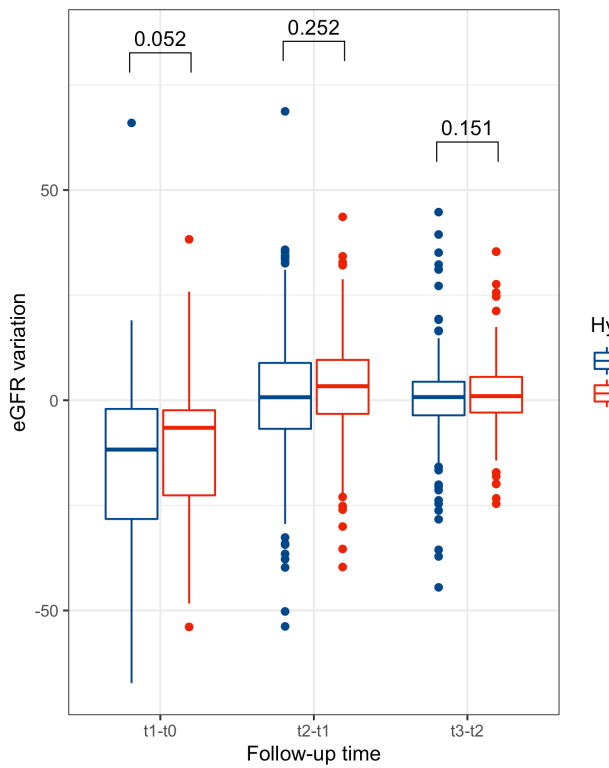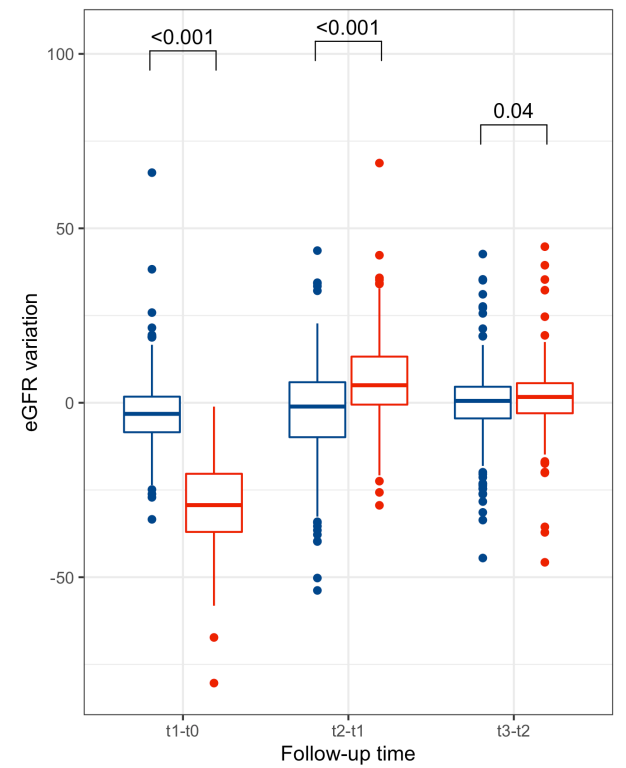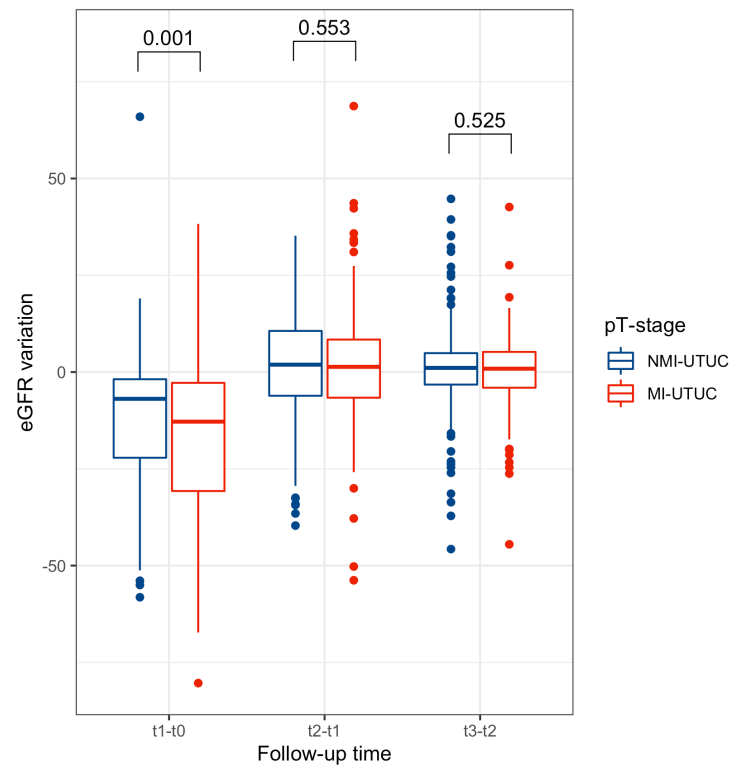

Supplement: Supplementary file 2 — Supplementary Fig. 2 eGFR variation according to clinically meaningful covariates. The eGFR variation was calculated as the difference between each timepoint and the previous timepoint (∆1 = eGFR POD I – eGFR preoperative; ∆2 = eGFR at 6 month – eGFR POD III, ∆3 = eGFR 12 month – eGFR 6 month). Differences between each group were evaluated with the Wilcoxon test after adjusting for multiple hypothesis testing according to the false discovery rate method (PDF 844 KB) [file 345_2022_4156_MOESM2_ESM.pdf]
