# Supplementary material for: Changes in renal function after nephroureterectomy for upper urinary tract carcinoma: analysis of a large multicenter cohort (Radical Nephroureterectomy Outcomes (RaNeO) Research Consortium)
Source: World J Urol. 2022 Oct 6;40(11):2771–9. doi: 10.1007/s00345-022-04156-3 (PMC9617815; doi:10.1007/s00345-022-04156-3)
Supplement: Supplementary file 3 — Supplementary file3 (DOCX 21 KB) [file 345_2022_4156_MOESM3_ESM.docx]

| **Supplementary Table 1** - Univariable and multivariable logistic regression models predicting I post-operative day acute kidney injury. | | |
| --- | --- | --- |
|  | **Univariable analysis** | **Multivariable analysis** |
|  | **Odds ratio (95%CI, p-value)** | **Odds ratio (95%CI, p-value)** |
| **Age** | 1.01 (0.99-1.03, p=0.330) | **1.04 (1.02-1.07, p<0.001)** |
| **Gender (male vs. female)** | **1.67 (1.15-2.44, p=0.008)** | 1.12 (0.72-1.74, p=0.614) |
| **BMI** | 0.99 (0.95-1.03, p=0.689) | - |
| Overweight vs. normal | 0.72 (0.42-1.23, p=0.232) | - |
| Obese vs. normal | 0.92 (0.61-1.39, p=0.690) | - |
| **ASA score (>2 vs. ≤2)** | 1.02 (0.71-1.47, p=0.895) | - |
| **Hydronephrosis (yes vs. no)** | **0.50 (0.35-0.73, p<0.001)** | **0.61 (0.39-0.93, p=0.022)** |
| **CAD** | 1.41 (0.91-2.19, p=0.122) | **1.89 (1.07-3.36, p=0.029)** |
| **Hypertension** | 1.18 (0.82-1.68, p=0.372) | - |
| **Hyperlipidemia** | 0.78 (0.54-1.11, p=0.170) | 0.70 (0.44-1.09, p=0.120) |
| **Diabetes** | 0.78 (0.51-1.20, p=0.262) | - |
| **Preoperatory eGFR** | **1.03 (1.02-1.04, p<0.001)** | **1.04 (1.03-1.05, p<0.001)** |
| **pT-stage (NMI vs. MI)** | **1.71 (1.21-2.43, p=0.003)** | **1.74 (1.14-2.67, p=0.010)** |
| **pN-stage** |  |  |
| pN1 vs. pN0 | 0.49 (0.21-1.07, p=0.087) | - |
| pN2 vs. pN0 | 1.84 (0.93-3.69, p=0.081) | - |
| pNx vs. pN0 | 1.23 (0.84-1.80, p=0.287) | - |
| **Tumor grade** |  |  |
| Low grade vs. High | 1.31 (0.79-2.20, p=0.297) | - |
