# Supplementary material for: Changes in renal function after nephroureterectomy for upper urinary tract carcinoma: analysis of a large multicenter cohort (Radical Nephroureterectomy Outcomes (RaNeO) Research Consortium)
Source: World J Urol. 2022 Oct 6;40(11):2771–9. doi: 10.1007/s00345-022-04156-3 (PMC9617815; doi:10.1007/s00345-022-04156-3)
Supplement: Supplementary file 4 — Supplementary file4 (DOCX 21 KB) [file 345_2022_4156_MOESM4_ESM.docx]

| **Supplementary Table 2 –** Multivariable linear mixed models predicting eGFR variation. The eGFR variation was calculated as the difference between each time-point and the previous timepoint (i.e. Time 1 = eGFR POD I – eGFR pre-operative; Time 2 = eGFR at 6 month – eGFR POD III). In bold are reported statistically significant results. | | |
| --- | --- | --- |
| **Covariates** | **Coefficient (± standard error)** | **p-value** |
| **Overall population** | | |
| **ECOG-PS (≤1 vs. >1)** | **- 2.5 (± 1.0)** | **0.024** |
| **Hyperlipidemia (yes vs. no)** | 1.5 (± 0.8) | 0.068 |
| **Positive surgical margin (yes vs. no)** | 1.1 (± 2.9) | 0.710 |
| **Hydronephrosis (yes vs. no)** | **9.2 (± 2.2)** | **<0.001** |
| **Tumor stage (NMI-UTUC vs. MI-UTUC)** | -1.9 (± 0.8) | 0.016 |
| **Age (≥75 vs. <75 years)** | -0.1 (± 0.9) | 0.875 |
| **Time** | **9.2 (± 0.7)** | **<0.001** |
| **Interaction Time: Hydronephrosis** | **-3.7 (± 0.9)** | **<0.001** |
| **Patients with AKI** | | |
| **Hydronephrosis (yes vs. no)** | 2.6 (±1.3) | **0.045** |
| **ASA Score (>2 vs. ≤2)** | 6.7 (±3.3) | **0.045** |
| **Age (≥75 vs. <75 years)** | 1.6 (±1.3) | 0.229 |
| **Time** | 16.9 (±1.0) | **<0.001** |
| **Interaction Time: ASA score** | -3.6 (±2.4) | **0.019** |
